# Association of longitudinal pet ownership with wheezing in 3-year-old children using the distributed lag model: the Japan Environment and Children’s Study

**DOI:** 10.1186/s12940-024-01087-x

**Published:** 2024-06-06

**Authors:** Kota Shirato, Koji Oba, Yutaka Matsuyama, Yasuhiro Hagiwara, The Japan Environment and Children’s Study (JECS) Group

**Affiliations:** 1 Department of Biostatistics, School of Health Sciences and Nursing, Graduate School of Medicine, the University of Tokyo, Bunkyo-ku, Tokyo, Japan; 2 Department of Biostatistics, School of Public Health, Graduate School of Medicine, the University of Tokyo, Bunkyo-ku, Tokyo, Japan

**Keywords:** Critical window, Distributed lag model, Japan, JECS, Pets, Time-varying exposure, Wheezing

## Abstract

**Background:**

Time-varying exposures like pet ownership pose challenges for identifying critical windows due to multicollinearity when modeled simultaneously. The Distributed Lag Model (DLM) estimates critical windows for time-varying exposures, which are mainly continuous variables. However, applying complex functions such as high-order splines and nonlinear functions within DLMs may not be suitable for situations with limited time points or binary exposure, such as in questionnaire surveys.

**Objectives:**

(1) We examined the estimation performance of a simple DLM with fractional polynomial function for time-varying binary exposures through simulation experiments. (2) We evaluated the impact of pet ownership on childhood wheezing onset and estimate critical windows.

**Methods:**

(1) We compared logistic regression including time-varying exposure in separate models, in one model simultaneously, and using DLM. For evaluation, we employed bias, empirical standard error (EmpSE), and mean squared error (MSE). (2) The Japan Environment and Children's Study (JECS) is a prospective birth cohort study of approximately 100,000 parent-child pairs, registered across Japan from 2011 to 2014. We applied DLM to the JECS data up to age 3. The estimated odds ratios (OR) were considered to be within critical windows when they were significant at the 5% level.

**Results:**

(1) DLM and the separate model exhibited lower bias compared to the simultaneously model. Additionally, both DLM and the simultaneously model demonstrated lower EmpSEs than the separate model. In all scenarios, DLM had lower MSEs than the other methods. Specifically, where critical windows is clearly present and exposure correlation is high, DLM showed MSEs about 1/2 to 1/200 of those of other models. (2) Application of DLM to the JECS data showed that, unlike other models, a significant exposure effect was observed only between the ages of 0 and 6 months. During that periods, the highest ORs were 1.07 (95% confidence interval, 1.01 to 1.14) , observed between the ages of 2 and 5 months.

**Conclusions:**

(1) A simple DLM improves the accuracy of exposure effect and critical windows estimation. (2) 0–6 months may be the critical windows for the effect of pet ownership on the wheezing onset at 3 years.

**Supplementary Information:**

The online version contains supplementary material available at 10.1186/s12940-024-01087-x.

## Introduction

Currently, proportion of both dog ownership and cat ownership in developed countries ranges from about 5% to 35% [1–3]. The expected effects of pets on children [4] include enhanced compassion [5], more time for activity [6], improved mobility [7] and learning [8], reduced pain [9] and stress [10], improved symptoms in children with disabilities [11], and less susceptibility to asthma and allergies [12]. Several systematic reviews have examined the impact of pet ownership on asthma and allergy development in children, but no consistent results have been obtained [13–15]. A 2011 systematic review of perinatal urban pet exposure and the development of asthma and allergy in children (9 studies through 2011) suggests that pet exposure may reduce allergy occurrence [13]. However, limitations exist, including differences in outcome measures and inconsistency in the effect of family history of allergy in each study. Additionally, the maximum sample size of the studies analyzed was only approximately 3,000; Given the possible influence of family history on allergies on the child’s allergies [13, 16], it is important to evaluate the association between pet ownership and asthma, wheezing, and other related symptoms using high-quality, large-size prospective studies based on the life course from before birth.

When evaluating the association between exposure and outcome based on the life course, the association may be expected to vary at different time points; the periods of exposure during which the association between exposure and outcome is strong are called the critical windows/ critical periods [17] (hereinafter, “critical windows”). Critical windows have also been noted with respect to pet ownership and asthma/wheezing in children. For example, the hygiene hypothesis, proposed in 1989 as a risk factor for asthma, is still being discussed [18, 19]. This hypothesis states that if people are exposed to allergens from an early age, they are less likely to develop allergic symptoms in the future due to immune tolerance [20, 21]. Accurately assessing the existence of critical windows and the extent of their effects will be useful for understanding the biological mechanisms associated with exposure and disease onset, as well as for preventing the latter [22].

A simple analysis estimating critical windows is to include exposures at each time point in a logistic regression model simultaneously. However, this cannot accurately assess the critical windows due to multicollinearity caused by high correlations among the explanatory variables [23]. Not only continuous but binary exposures, such as pet ownership, are also known to be suffered from multicollinearity [24]. Various statistical methods has been proposed for estimating critical windows under the situation [22, 23, 25–29]. In environmental epidemiology, the Distributed Lag Model (DLM) is an alternative analytical model to evaluate the time-dependent association between time-varying exposures and outcome at a specific time point [22, 23, 25–27]. On the other hand, within the DLM framework, there has been a growing trend towards the utilization of complicated functions, including high-order splines and nonlinear functions, to describe a smooth relationship between time-varying exposures and the outcome [23]. However, it is not a good strategy to apply such complicated functions to DLMs in situations with limited time points or binary exposure, such as in questionnaire surveys. For instance, when employing splines in DLM, the implementation would include estimating random effects [30]. However, there are a couple of challenges: (1) A limited number of measurements can lead to computational constraints and diminish the advantages of introducing knots, and (2) using an overly flexible model may result in being overly influenced by data noise, making it difficult to interpret critical windows (e.g., beneficial periods not being consecutive [22]).

In this paper, we propose a simple DLM with fractional polynomial function to evaluate the critical windows with respect to pet ownership and asthma/wheezing in children. This approach, applicable in most standard statistical software only through variable transformation, is as flexible as possible while remaining simple and easy to understand. The purpose of our study is to examine the estimation performance of the proposed logistic regression model with a time-varying binary exposure through simulation experiments and estimate the effect and critical windows of exposure to pets on childhood wheezing by applying DLM to the Japan Environment and Children’s Study (JECS) data of 100,000 pairs of children and their parents.

## Methods

### Application data

The JECS is an ongoing birth cohort study of approximately 100,000 pairs of children and their parents registered between January 2011 and March 2014, aimed at clarifying how exposure to chemical substances and the living environment affects children's health from fetal stage through childhood; the study aims to establish an appropriate risk management system [31]. In the JECS, participants are recruited from 15 Regional Centres across Japan (Hokkaido, Miyagi, Fukushima, Chiba, Kanagawa, Koshin, Toyama, Aichi, Kyoto, Osaka, Hyogo, Tottori, Kochi, Fukuoka, and South Kyushu/Okinawa), and data on exposure to chemical substances, environmental factors other than chemical substances (e.g., house dust), genetic factors, and social factors are collected as exposures. Additionally, the JECS collects information on early life growth and development (e.g., developmental status in childhood and disorders of the immune system and metabolism) as outcomes. The JECS Protocol was reviewed and approved by the Ministry of Environment’s Institutional Review Board on Epidemiological Studies and by the Ethics Committees of all participating institutions. Written informed consent was obtained from all participants. The JECS was conducted in accordance with the principles laid out in the Declaration of Helsinki and other national regulations and guidelines. Our study was conducted using fixed data (jecs-ta-20190930) up to 3 years after birth in the JECS after approval from the Steering Committee of the JECS.

**Fig. 1:**
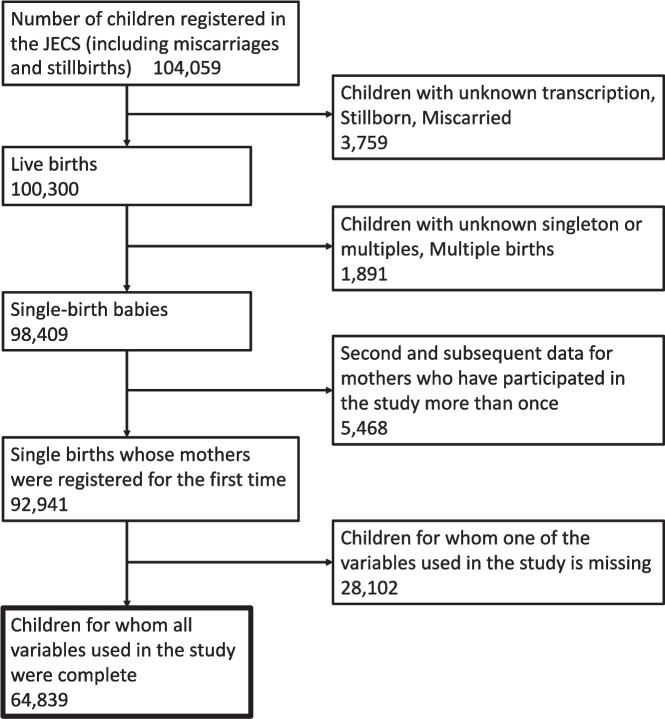
Subject selection flowchart. The numbers correspond to the number of people

The study sample comprised 64,839 participants, among the 92,941 singleton births from mothers who were registered for the first time in the JECS, upon meeting the following conditions: 1) all exposure, time point, covariate, and outcome data were measured, and 2) the study time points were not contradictory, specifically, with the 3-year-old’s questionnaire as the reference point, the 1.5-year-old’s questionnaire's response time ranged from 6 to 30 months earlier, the 6-month-old’s questionnaire's response time ranged from 18 to 42 months earlier, and the mid-pregnancy questionnaire's response time ranged from 27 to 51 months earlier. Figure 1 shows the flowchart of the selection of the study sample from among JECS participants.

For the variables used, we referred to a study examining the associations of pet ownership with wheezing and asthma in children in the Pilot Study of JECS [32]. For variables with category classification differing from the that in the Pilot Study, we referred to previous studies that used data from the JECS Main Study and used asthma as an outcome [33–36].

Time-varying exposure was pet (dog or cat) ownership, measured thrice using a questionnaire: at mid-pregnancy (mean gestational age at the time of questionnaire response = 27.9 weeks [37]) and when the child was 6 months and 1.5 years old. The primary outcome among the variables available as asthma-related outcomes in the 3-year-old’s questionnaire was the presence of wheezing in the last 12 months (“Has your child had wheezing or whistling in the chest in the past 12 months?”). This question was partially modified from the International Study of Asthma and Allergies in Childhood (ISAAC) questionnaire for 6–7-year-olds, the translation of which has been validated in Japanese [38–40]. Other outcome options included physicians’ diagnosis of asthma (“Has your child ever been diagnosed as follows by a physician?” _ Immune system disorder diagnosed after age 2: Asthma), experience with asthma (“Has your child ever had asthma?”), and experience with wheezing (“Has your child ever had wheezing or whistling in the chest at any time in the past?”). In this study, only the presence of wheezing in the last 12 months was used as an outcome, as the time of wheezing onset was later than the time of the exposure questionnaire, and it is difficult to diagnose asthma clinically in 3-year-olds [41]. The variables used for confounding adjustment as baseline covariates were as follows: previous delivery (categorized as yes, no), weeks of pregnancy at delivery (premature birth [22–36 weeks], full-term birth [37–41 weeks], other), planned/emergent cesarean delivery (yes, no), weight at birth (< 2,500 g, ≥ 2,500 g), child’s sex at birth (male, female, indeterminate), annual household income (< 4, ≥ 4–6, ≥ 6 million JPY), frequency of cleaning the living room floor with a vacuum cleaner (average throughout the year; categorized as every day, once a week and more, less than once a week), frequency of cleaning the bedroom floor with a vacuum cleaner (average throughout the year; categorized as every day, once a week and more, less than once a week), family members' smoking after the baby was born within 1 month of birth (no one smoked, somebody smoked but not in the presence of the baby, somebody smoked in the presence of the baby), mother’s allergy and ear-nose-throat disease (bronchial asthma; yes, no), and Study Areas (Additional file 1, Table A1).

The JECS questionnaire is administered in the month when the child reaches the target age (that is, 3 years 0 months, 1.5 years 0 months, and 6 months). We summarized the actual time point for each questionnaire (subtracted from the time point of the 3-year-old’s questionnaire).

#### Statistical analysis

In this paper, the following notations are used: $i (i=1,\dots ,n)$ for the participant identification number, ${Y}_{i}$ for the presence of the outcome at the 3-year-old’s questionnaire $(1\text{ if present}, 0\text{ if not})$; $q (q=18, 30, 39)$ for the time points representing 1.5-years-old, 6-months-old, and mid-term pregnancy; ${t}_{{18}_{i}}{ , t}_{{30}_{i}}{ , t}_{{39}_{i}}$ indicating how many months before the response to the 3-year-old’s questionnaire the responses corresponding to 1.5-year-old’s questionnaire, 6-month-old’s questionnaire, and mid-pregnancy questionnaire were obtained; ${X}_{{q}_{i}}$ indicating whether or not respondents had pets corresponding to each time point; ${\pi }_{i}$ denoting the probability of occurrence of the outcome ${Y}_{i}$; ${\beta }_{q}$ for the regression coefficient on exposure, the regression coefficient vector ${{\varvec{\eta}}}_{{\varvec{q}}}^{{\varvec{T}}}$ for the baseline covariates and the baseline covariates vector ${{\varvec{Z}}}_{{\varvec{i}}}$. The regression model considered in this study is described next.

### Regression model for each time point (Single Model)

The “Single Model” is the simplest analysis of time-varying exposure, where the exposure at each time point is used in a separate regression model. As we have three time points of exposure to consider, we evaluate the following three regression models separately.

$$\begin{array}{c}{\text{log}}\left(\frac{{\pi }_{i}}{1-{\pi }_{i}}\right)={\beta }_{{0}_{1}}+{\beta }_{18}{X}_{18i}+{{\varvec{\eta}}}_{18}^{{\varvec{T}}}{{\varvec{Z}}}_{{\varvec{i}}}\\ {\text{log}}\left(\frac{{\pi }_{i}}{1-{\pi }_{i}}\right)={\beta }_{{0}_{2}}+{\beta }_{30}{X}_{30i}+{{\varvec{\eta}}}_{30}^{{\varvec{T}}}{{\varvec{Z}}}_{{\varvec{i}}}\\ {\text{log}}\left(\frac{{\pi }_{i}}{1-{\pi }_{i}}\right)={\beta }_{{0}_{3}}+{\beta }_{39}{X}_{39i}+{{\varvec{\eta}}}_{39}^{{\varvec{T}}}{{\varvec{Z}}}_{{\varvec{i}}}\end{array}$$

### Regression model including all time points of exposure simultaneously (Multi Model)

In this model, the exposures at each time point are simultaneously entered into a single regression model.

1 $${\text{log}}\left(\frac{{\pi }_{i}}{1-{\pi }_{i}}\right)={\beta }_{0}+{\beta }_{18}{X}_{18i}+{\beta }_{30}{X}_{30i}+{\beta }_{39}{X}_{39i}+{{\varvec{\eta}}}^{{\varvec{T}}}{{\varvec{Z}}}_{{\varvec{i}}}$$

### Distributed Lag Model(DLM)

When considering that past exposures ${X}_{18i} ,{X}_{30i} ,{X}_{39i}$ have effects on outcome ${Y}_{i}$, the unconstrained DLM, is the same as the Multi Model. To address the correlations among ${X}_{18i} ,{X}_{30i} ,{X}_{39i}$, we consider constraining the regression coefficients ${\beta }_{q}$ as a polynomial function of ${t}_{qi}$, where each is measured as follows:

2 $${\beta }_{{\text{q}}}=f\left({t}_{qi}\right)={\delta }_{0}+{\delta }_{1}{t}_{qi}+{\delta }_{2}{t}_{qi}^{2}+ {\delta }_{3}\sqrt{{t}_{qi}}$$

Substituting eq. (2) into eq. (1), we get

$$\begin{array}{c}{{\text{log}}\left(\frac{{\pi }_{i}}{1-{\pi }_{i}}\right)=\beta }_{0}+f\left({t}_{{18}_{i}}\right){X}_{18i}+f\left({t}_{{30}_{i}}\right){X}_{30i}+f\left({t}_{{39}_{i}}\right){X}_{39i}+{{\varvec{\eta}}}^{{\varvec{T}}}{{\varvec{Z}}}_{{\varvec{i}}}\\ ={\beta }_{0}+{{\varvec{\delta}}}^{{\varvec{T}}}{{\varvec{W}}}_{{\varvec{i}}}+{{\varvec{\eta}}}^{{\varvec{T}}}{{\varvec{Z}}}_{{\varvec{i}}}\end{array}$$

where

$${{\varvec{W}}}_{{\varvec{i}}}=\left[\begin{array}{c}{X}_{18i}+{X}_{30i}+{X}_{39i}\\ {t}_{{18}_{i}}{X}_{18i}+{t}_{{30}_{i}}{X}_{30i}+{t}_{{39}_{i}}{X}_{39i}\\ {t}_{{18}_{i}}^{2}{X}_{18i}+{t}_{{30}_{i}}^{2}{X}_{30i}+{t}_{{39}_{i}}^{2}{X}_{39i}\\ \sqrt{{t}_{{18}_{i}}}{X}_{18i}+\sqrt{{t}_{{30}_{i}}} {X}_{30i}+\sqrt{{t}_{{39}_{i}}} {X}_{39i}\end{array}\right]$$

Next, we compute the regression coefficients in eq. (3) using the maximum likelihood estimation method.

3 $${\text{log}}\left(\frac{{\pi }_{i}}{1-{\pi }_{i}}\right)={\beta }_{0}+{{\varvec{\delta}}}^{{\varvec{T}}}{{\varvec{W}}}_{{\varvec{i}}}+{{\varvec{\eta}}}^{{\varvec{T}}}{{\varvec{Z}}}_{{\varvec{i}}}$$

Since we are interested in the effect of exposure ${\beta }_{q}\text{ at time }q,\text{ we estimate the exposure effect }\widehat{{\beta }_{q}}=\widehat{f({t}_{qi})}\text{ by substituting the }\widehat{{\delta }_{0}} , \widehat{{\delta }_{1}} , \widehat{{\delta }_{2}} , \widehat{{\delta }_{3}}$ obtained from eq. (3) into eq. (2).

The variance of the estimator $\widehat{{\beta }_{{\text{q}}}}$ is calculated using the covariance of $$\widehat{{\varvec{\delta}}}\text{ } \left(Cov\left(\widehat{{\delta }_{0}} , \widehat{{\delta }_{1}} , \widehat{{\delta }_{2}} , \widehat{{\delta }_{3}}\right)\right)\text{ and }{{\varvec{T}}}_{{\varvec{q}}}={\left[\begin{array}{cc}\begin{array}{cc}1& {t}_{qi}\end{array}& \begin{array}{cc}{t}_{qi}^{2}& \sqrt{{t}_{qi}}\end{array}\end{array}\right]}^{{\varvec{T}}}$$ in the following equation.

$$V\left(\widehat{f({t}_{qi})}\right)={{\varvec{T}}}_{{\varvec{q}}}^{{\varvec{T}}}Cov\left(\widehat{{\delta }_{0}} , \widehat{{\delta }_{1}} , \widehat{{\delta }_{2}} , \widehat{{\delta }_{3}}\right){{\varvec{T}}}_{{\varvec{q}}}$$

We call eq. (3) the DLM.

In all models, a time point was considered a critical window when the estimated odds ratio was statistically significant at a two-sided significance level of 5%.

#### Simulation experiments

##### Data generation

To evaluate the statistical performance of the DLM, we performed simulation experiments in the following settings.

- Time point

For the number of time-varying exposures, we considered having the respondents answer the questionnaire 10 times. We considered a variation for a time point of exposures. The distribution of time points in the $k\left(k=1, \dots , 10\right)\text{ th questionnaire was generated from }p\left({t}_{ki}=6k\right)=0.45, p\left({t}_{ki}=6k\pm 1\right)=0.15, p\left({t}_{ki}=6k\pm 2\right)=0.10, p\left({t}_{ki}=6k+3\right)=0.05$ (Additional file 1, Table B1).

- Binary exposures

To correlate the binary longitudinal exposures ${X}_{{t}_{ki}}\text{ at individual }i$with each other, the following procedure was used to generate the exposure data [42, 43].

1. Determine the probability ${p}_{k}$ of having exposure at each time point. In this study, all probabilities were set as 0.2.

$$P\left({X}_{{t}_{ki}}=1\right)=0.2={p}_{k}$$

2. Set up a matrix $\mathbf{C}$ that determines the magnitude of the correlation.

$$\mathbf{C}=\left(\begin{array}{ccc}{c}_{11}& \cdots & {c}_{1, 10}\\ \vdots & \ddots & \vdots \\ {c}_{\mathrm{10,1}}& \cdots & {c}_{\mathrm{10,10}}\end{array}\right)$$

A first-order autoregressive (AR(1)) model was assumed for $$\mathbf{C},\text{ with }{c}_{ij}=\left\{\begin{array}{c}1 (i=j)\\ {\gamma }^{|i-j|} (i \ne j)\end{array}\right.$$. For γ, we used γ = 0.975, which assumes the JECS data, and added γ = 0, 0.9999 for comparison, for a total of three types.

3. We generate 10 variables ${\varvec{V}}=({V}_{1}, {V}_{2} , \dots , {V}_{10})$ that follow the multivariate normal distribution below.

$${\varvec{V}}\boldsymbol{ }\sim \boldsymbol{ }\mathcal{N}(0,\boldsymbol{ }\mathbf{C})$$

4. ${\varvec{V}},\boldsymbol{ }{p}_{k},$ the inverse function $g\left(\bullet \right)$ of the cumulative distribution function of the normal distribution is used to generate ${\varvec{X}}=({X}_{{t}_{1i}} , {X}_{{t}_{2i}} , \dots , {X}_{{t}_{10i}})$.
  $$\left\{\begin{array}{c}{X}_{{t}_{ki}}=1 ({V}_{k}<g({p}_{k}))\\ {X}_{{t}_{ki}}=0 (otherwise)\end{array}\right.$$

- Number of participants in each dataset

*N*=10,000 data sets were generated for each of γ=0 , 0.975, and 0.9999.

- True exposure effects

**Fig. 2:**
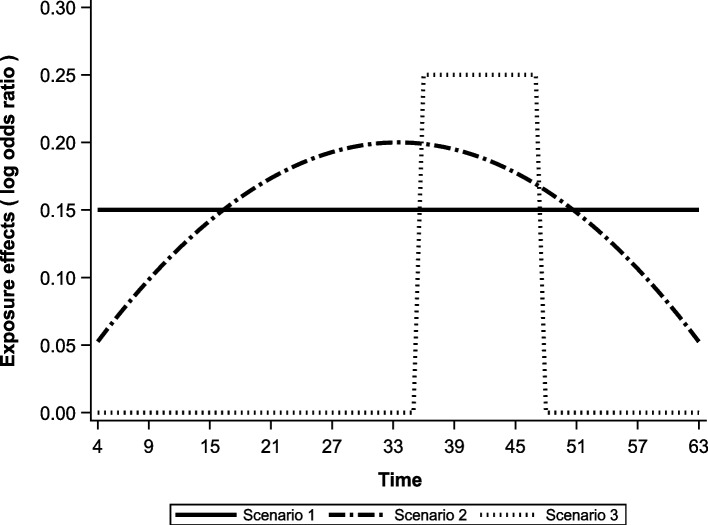
True value scenario for β(t) used in the simulation experiment. Scenario 1: Exposure effects are constant regardless of time point. Scenario 2: Exposure effects’ magnitude is an upward convex quadratic curve. Scenario 3: Exposure effects exist only from $t = 36 \text{ to } 47$

We consider the following three patterns for the true exposure effects $\beta \left(t\right)$ (Fig. 2).

1. Exposure effects are constant regardless of time point ($\beta \left(t\right)=0.15$).
2. Exposure effects always exist, and the magnitude is an upward convex quadratic curve.
3. Exposure effects exist only from $t = 36 \text{ to } 47$

For scenario (2), the true value $\beta \left(t\right)$ is generated by a quadratic function of time. Scenario (3) is the scenario in which the analytical model described above misspecifies the true association between exposure and outcome.

- Outcome

The probability of occurrence of outcome $P({y}_{i}=1)$ (asthma-related outcomes in the 3-year-old’s questionnaire) is set to approximately 10% in accordance with the incidence of asthma in the JECS. The following formula with an intercept of -2.2 is used to calculate the true value of the probability of occurrence ${\mu }_{i}$, and the individual outcome ${y}_{i}$ is generated from the Bernoulli distribution based on ${\mu }_{i}$.

$$\begin{array}{c}{\mu }_{i}=-2.2+\sum_{k=1}^{10}{X}_{{t}_{ki}}\beta \left(k\right) \left(i=1, \dots , N \right)\\ {y}_{i}\sim Bernoulli\left(\frac{{\text{exp}}\left({\mu }_{i}\right)}{1+{\text{exp}}\left({\mu }_{i}\right)}\right) \left(i=1, \dots , N\right)\end{array}$$

##### Performance measures

Single Model, Multi Model, and DLM were applied to the simulation experiment data. The simulation experiments were repeated 1000 times for each scenario and correlation coefficient γ. The performance measurements to assess the performance of each analytical model included bias $\left(E\left[\widehat{\beta \left(k\right)}\right]-\beta \left(k\right)\right)$, empirical standard error $\left(EmpSE; \sqrt{Var(\widehat{\beta \left(k\right)})}\right)$, and mean squared error $\left(MSE; E\left[{\left\{\widehat{\beta \left(k\right)}-\beta \left(k\right)\right\}}^{2}\right]\right)$.

SAS® 9.4 (SAS Institute Inc., Cary, North Carolina) was used for all statistical analyses, including simulation experiments and real data analysis.

## Results

### Simulation experiments

#### Magnitude of bias for exposure effect estimates

The Single Model estimated the exposure effect without bias when the correlation between exposures was zero, but when the correlation between exposures was high, it showed a large bias, predominantly from the null. The Multi Model also showed an increase in bias, both toward and away from the null, as the correlation between exposures increased, but the magnitude of the bias was stable and small throughout all scenarios. The DLM also showed the same trend of increasing bias, both toward and away from the null, as the correlation between exposures increased, with low bias in Scenarios 1 and 2, but a bias of approximately 50% of the true value in critical windows in Scenario 3 (Table 1).

**Table 1:** Simulation experiment results: bias

time	γ=0			γ=0.975			γ=0.9999		
	Single	Multi	DLM	Single	Multi	DLM	Single	Multi	DLM
Scenario 1									
6	0.0	0.1	0.0	93.2	-0.2	0.1	131.7	1.4	-0.8
12	-0.3	-0.2	-0.1	97.3	0.5	-0.1	131.9	-3.1	-0.5
18	-0.5	-0.4	-0.1	100.1	-0.4	-0.1	132.1	0.2	-0.2
24	0.1	0.2	-0.1	101.9	0.0	-0.1	132.3	-0.1	0.1
30	-0.3	-0.2	-0.1	102.8	0.2	-0.1	132.3	2.1	0.3
36	-0.2	-0.1	-0.1	102.7	-1.6	-0.1	132.3	0.0	0.4
42	-0.3	-0.3	-0.1	102.2	1.1	0.0	132.3	0.6	0.3
48	-0.1	0.0	-0.1	100.3	-0.1	0.1	132.1	-1.7	0.2
54	-0.1	-0.1	-0.1	97.6	1.1	0.2	131.9	0.0	0.0
60	-0.3	-0.2	-0.1	93.4	-0.5	0.3	131.7	0.0	-0.4
Scenario 2									
6	0.4	0.3	0.1	98.9	-0.2	-0.1	139.4	-1.2	-0.1
12	-0.3	-0.2	-0.2	99.1	0.1	-0.1	134.8	3.4	-0.2
18	-0.4	-0.3	-0.2	99.7	-0.1	0.0	131.3	-0.6	-0.2
24	-0.3	-0.2	-0.2	100.3	0.4	0.1	129.0	0.5	-0.1
30	-0.3	-0.2	-0.1	100.6	-0.1	0.2	127.7	-3.1	-0.1
36	0.0	0.1	0.0	100.7	1.1	0.2	127.6	-2.5	0.0
42	-0.3	-0.2	0.0	100.1	-0.8	0.1	128.7	3.0	0.0
48	-0.2	-0.1	0.0	99.4	-0.3	0.0	130.8	2.3	0.1
54	-0.5	-0.4	0.0	98.7	-0.3	-0.2	134.1	-3.2	0.2
60	-0.1	0.0	0.0	98.3	-0.4	-0.4	138.6	1.1	0.3
Scenario 3									
6	0.3	0.3	0.9	33.6	0.5	-0.1	49.4	0.9	-3.4
12	0.4	0.4	-4.2	35.1	0.5	-4.1	49.5	0.3	-3.2
18	-0.3	-0.3	-0.9	36.6	-0.9	-0.6	49.6	-1.6	0.9
24	-0.1	-0.1	4.5	38.7	-0.1	4.6	49.8	-0.7	5.6
30	-0.1	-0.1	9.4	41.3	0.4	9.4	50.0	2.3	9.4
36	-6.5	-6.5	-12.3	20.8	-5.9	-12.4	25.2	-5.8	-13.2
42	-0.4	-0.4	-11.4	21.9	-0.6	-11.6	25.2	-7.5	-12.8
48	6.5	6.6	11.5	43.1	6.3	11.4	50.1	9.2	10.5
54	0.2	0.2	6.2	39.9	0.1	6.3	49.9	3.4	6.5
60	-0.5	-0.5	-2.6	37.7	0.3	-2.1	49.7	-0.3	-0.1

Bias rounded to the fourth decimal place and multiplied by ${10}^{2}$

Single, the exposure at each time point is used in a separate regression model; Multi, the exposures at each time point are simultaneously entered into a single regression model; DLM, the Distributed Lag Model

γ represents the correlation used to generate data between exposures

Scenario 1: Exposure effects are constant regardless of time point. Scenario 2: Exposure effects’ magnitude is an upward convex quadratic curve. Scenario 3: Exposure effects exist only from $t = 36 \text{ to } 47$

The means of the point estimates of the exposure effects estimated by DLM in the simulation experiments are shown in Additional file 1, Figure B1 (for each scenario and correlation coefficient γ). In the DLM, in Scenarios 1 and 2, the mean of the point estimates matched the shape of the true $\beta \left(k\right)$. In Scenario 3, the shape did not match, but the peak of the true value was captured.

#### Magnitude of empirical standard errors for exposure effect estimates

The standard errors for the exposure effect estimates by the Single Model were generally in the range of 0.06–0.08 for all scenarios, regardless of the correlation γ. The empirical standard errors for the Multi Model and DLM tended to increase as the correlation increased; the Multi Model was more pronounced, approximately twice as large as the Single Model for data generated at γ= 0.975. The DLM exhibited a smaller increase in empirical standard errors than the Multi Model. Specifically, at certain time points with γ= 0.975 (Table 2), the DLM had the smallest standard errors among the three models. This feature was also confirmed by the box-and-whisker plot (Additional file 1, Figures B2-B4).

**Table 2:** Simulation experiment results: empirical squared error

time	γ=0			γ=0.975			γ=0.9999		
	Single	Multi	DLM	Single	Multi	DLM	Single	Multi	DLM
Scenario 1									
6	6.9	7.0	6.4	6.1	12.5	8.2	6.1	51.7	18.2
12	7.1	7.2	4.4	6.1	15.2	4.4	6.0	61.3	12.1
18	7.0	7.1	4.2	6.1	15.4	4.3	6.1	62.3	9.2
24	7.4	7.4	3.6	6.0	14.9	3.5	6.1	61.5	7.8
30	7.2	7.3	3.4	6.0	14.8	3.4	6.1	60.0	8.4
36	7.4	7.3	3.7	6.1	14.9	3.9	6.1	63.6	9.2
42	7.4	7.4	3.8	6.0	15.3	4.0	6.1	63.4	9.1
48	7.1	7.1	3.5	6.0	15.1	3.1	6.1	59.4	8.4
54	7.2	7.2	3.6	6.2	15.6	3.2	6.1	62.7	10.2
60	6.9	7.0	5.7	6.0	12.4	7.1	6.1	51.9	17.5
Scenario 2									
6	7.2	7.2	6.7	6.5	12.8	8.5	6.1	51.3	17.4
12	7.2	7.3	4.4	6.3	14.6	4.3	6.1	61.8	12.0
18	7.2	7.2	4.2	6.3	14.9	4.4	6.1	61.0	9.2
24	7.0	7.0	3.6	6.3	15.7	3.6	6.1	62.4	7.7
30	7.2	7.2	3.4	6.4	15.2	3.4	6.1	61.7	8.4
36	7.2	7.2	3.7	6.3	15.3	3.8	6.1	64.9	9.4
42	7.1	7.1	3.8	6.3	15.2	3.8	6.1	61.6	9.3
48	6.8	6.8	3.5	6.3	15.0	3.1	6.1	63.2	8.3
54	7.5	7.6	3.9	6.3	15.2	3.3	6.0	62.5	9.8
60	7.4	7.4	6.3	6.4	12.6	7.2	6.0	50.8	17.4
Scenario 3									
6	8.2	8.2	7.6	7.3	14.6	9.8	7.5	56.9	21.4
12	8.0	8.0	5.1	7.3	16.9	5.1	7.5	67.9	14.5
18	8.1	8.1	4.8	7.4	17.2	5.2	7.5	67.1	11.1
24	7.8	7.8	4.0	7.4	17.4	4.2	7.4	69.4	9.7
30	7.9	7.9	3.7	7.5	17.6	4.0	7.5	68.9	10.6
36	7.6	7.6	4.0	7.3	17.4	4.6	7.5	68.3	11.6
42	7.8	7.8	4.1	7.1	17.5	4.6	7.5	67.9	11.3
48	7.9	8.0	3.9	7.2	17.6	3.6	7.4	67.7	10.0
54	8.0	8.1	4.2	7.3	16.9	3.7	7.4	67.5	12.4
60	8.2	8.2	6.7	7.2	14.4	8.5	7.4	56.5	22.2

Empirical standard errors rounded to the fourth place and multiplied by ${10}^{2}$

Single, the exposure at each time point is used in a separate regression model; Multi, the exposures at each time point are simultaneously entered into a single regression model; DLM, the Distributed Lag Model

γ represents the correlation used to generate data between exposures.

Scenario 1: Exposure effects are constant regardless of time point. Scenario 2: Exposure effects’ magnitude is an upward convex quadratic curve. Scenario 3: Exposure effects exist only from $t = 36 \text{ to } 47$

#### Mean squared error

Table 3 shows the magnitude of the MSE, where the bias and standard error of the exposure effect estimates were evaluated simultaneously. When the correlation between exposures was high, the MSE increased for the Single Model and Multi Model. In almost all cases, DLM had the smallest MSE. Specifically, where CW is clearly present (scenario 3) and exposure correlation is high (γ= 0.975 and 0.9999), DLM showed MSEs about 1/2 to 1/200 of those of other models.

**Table 3:** Simulation experiment results: mean squared error

	γ=0			γ=0.975			γ=0.9999		
time	Single	Multi	DLM	Single	Multi	DLM	Single	Multi	DLM
Scenario 1									
6	0.5	0.5	0.4	87.3	1.6	0.7	173.8	26.7	3.3
12	0.5	0.5	0.2	95.1	2.3	0.2	174.4	37.7	1.5
18	0.5	0.5	0.2	100.5	2.4	0.2	174.9	38.8	0.8
24	0.6	0.6	0.1	104.3	2.2	0.1	175.3	37.8	0.6
30	0.5	0.5	0.1	106.1	2.2	0.1	175.5	36.1	0.7
36	0.5	0.5	0.1	105.8	2.2	0.2	175.5	40.4	0.9
42	0.5	0.5	0.1	104.7	2.4	0.2	175.3	40.1	0.8
48	0.5	0.5	0.1	100.9	2.3	0.1	174.9	35.3	0.7
54	0.5	0.5	0.1	95.7	2.4	0.1	174.5	39.2	1.0
60	0.5	0.5	0.3	87.6	1.5	0.5	173.8	26.9	3.1
Scenario 2									
6	0.5	0.5	0.5	98.2	1.6	0.7	194.8	26.3	3.0
12	0.5	0.5	0.2	98.7	2.1	0.2	182.2	38.3	1.4
18	0.5	0.5	0.2	99.8	2.2	0.2	172.8	37.2	0.8
24	0.5	0.5	0.1	101.1	2.4	0.1	166.7	38.9	0.6
30	0.5	0.5	0.1	101.7	2.3	0.1	163.5	38.1	0.7
36	0.5	0.5	0.1	101.8	2.3	0.1	163.3	42.1	0.9
42	0.5	0.5	0.1	100.5	2.3	0.1	166.0	38.0	0.9
48	0.5	0.5	0.1	99.2	2.2	0.1	171.6	40.0	0.7
54	0.6	0.6	0.2	97.9	2.3	0.1	180.2	39.1	1.0
60	0.5	0.6	0.4	97.1	1.6	0.5	192.3	25.7	3.0
Scenario 3									
6	0.7	0.7	0.6	11.8	2.1	1.0	25.0	32.4	4.7
12	0.6	0.6	0.4	12.9	2.8	0.4	25.1	46.1	2.2
18	0.7	0.7	0.2	13.9	3.0	0.3	25.2	45.1	1.2
24	0.6	0.6	0.4	15.5	3.0	0.4	25.3	48.2	1.2
30	0.6	0.6	1.0	17.6	3.1	1.1	25.5	47.5	2.0
36	1.0	1.0	1.7	4.8	3.4	1.8	6.9	47.0	3.1
42	0.6	0.6	1.5	5.3	3.1	1.6	6.9	46.6	2.9
48	1.1	1.1	1.5	19.1	3.5	1.4	25.6	46.6	2.1
54	0.6	0.6	0.6	16.5	2.8	0.5	25.5	45.6	2.0
60	0.7	0.7	0.5	14.8	2.1	0.8	25.3	31.9	4.9

MSE rounded to the fourth decimal place and multiplied by ${10}^{2}$

Single, the exposure at each time point is used in a separate regression model; Multi, the exposures at each time point are simultaneously entered into a single regression model; DLM, the Distributed Lag Model

γ represents the correlation used to generate data between exposures

Scenario 1: Exposure effects are constant regardless of time point. Scenario 2: Exposure effects’ magnitude is an upward convex quadratic curve. Scenario 3: Exposure effects exist only from $t = 36 \text{ to } 47$

### JECS data

#### Demographics and baseline characteristics

Table 4 shows participant demographics and baseline characteristics. The percentage of respondents with pets was approximately 10–20% at any time point. The categories of variables chosen by less than 10% of the total respondents were preterm birth, birth weight < 2,500 g, someone smoked even in the presence of the baby, and frequency of cleaning the living room being less than once a week. The number of participants from each Study Area varied from 2,716 (Kyoto) to 8,295 (Fukushima).

**Table 4:** Baseline characteristics and Percentage of wheezing incidence for each category

		Wheezing in the last 12 months		
			yes	no
variables	category	people	%	%
Mid pregnancy pet keeping	No	55538	17.1	82.9
	Yes	9301	18.9	81.1
6 months old pet keeping	No	53712	17.1	82.9
	Yes	11127	18.7	81.3
1.5 years old pet keeping	No	56740	17.1	82.9
	Yes	8099	19.0	81.0
Child’s sex at birth	Male	33161	19.7	80.3
	Female	31678	14.9	85.1
Weeks of pregnancy at delivery	Premature birth (22-36 weeks)	2782	23.0	77.0
	Full-term birth (37-41 weeks)	61900	17.1	82.9
	Others	157	16.6	83.4
Previous delivery	No	28588	15.2	84.8
	Yes	36251	19.1	80.9
Weight at birth	< 2,500 g	4980	19.3	80.7
	$\ge$ 2,500 g	59859	17.2	82.8
Family members' smoking after the baby was born within 1 month of birth	No smoking	32741	16.3	83.7
	Smoking in the absence of babies	30734	18.4	81.6
	Smoking in the presence of the baby	1364	19.9	80.1
Planned/emergent cesarean delivery	No	52853	17.1	82.9
	Yes	11986	18.6	81.4
Annual household income	<4 million JPY	24896	17.9	82.1
	4 - 6 million JPY	21861	17.0	83.0
	$\ge$ 6 million JPY	18082	17.1	82.9
Frequency of cleaning the living room floor	Every day	10727	17.1	82.9
	Once a week and over	48867	17.5	82.5
	Less than once a week	5245	16.5	83.5
Frequency of cleaning the bedroom floor	Every day	6709	16.9	83.1
	Once a week and over	49055	17.5	82.5
	Less than once a week	9075	17.2	82.8
Mother’s allergy and ear-nose-throat disease	Bronchial asthma; no	58026	15.9	84.1
	Bronchial asthma; yes	6813	29.9	70.1
Study Areas	Aichi	3522	13.2	86.8
	Miyagi	5335	16.4	83.6
	Kyoto	2716	19.4	80.6
	Koshin	4526	16.3	83.7
	Kochi	4231	17.3	82.7
	Kanagawa	4332	15.9	84.1
	Chiba	3622	16.6	83.4
	Osaka	5304	12.7	87.3
	Tottori	1851	21.5	78.5
	South Kyushu/Okinawa	3607	29.2	70.8
	Toyama	3852	15.3	84.7
	Fukuoka	5110	17.8	82.2
	Fukushima	8295	19.2	80.8
	Hyogo	3478	13.8	86.2
	Hokkaido	5058	18.4	81.6

At all time points, the pet-owning group had a higher incidence proportion of wheezing in the last 12 months than the non-pet-owning group (approximately 19% and 17%, respectively). Differences in wheezing incidence were also related to the child’s sex (male > female), premature birth status (premature > full term), and maternal asthma status (asthma > no asthma). By area, the highest wheezing incidence proportion was observed in South Kyushu/Okinawa (29.2%), and the lowest in Osaka (12.7%), indicating regional differences.

**Fig. 3:**
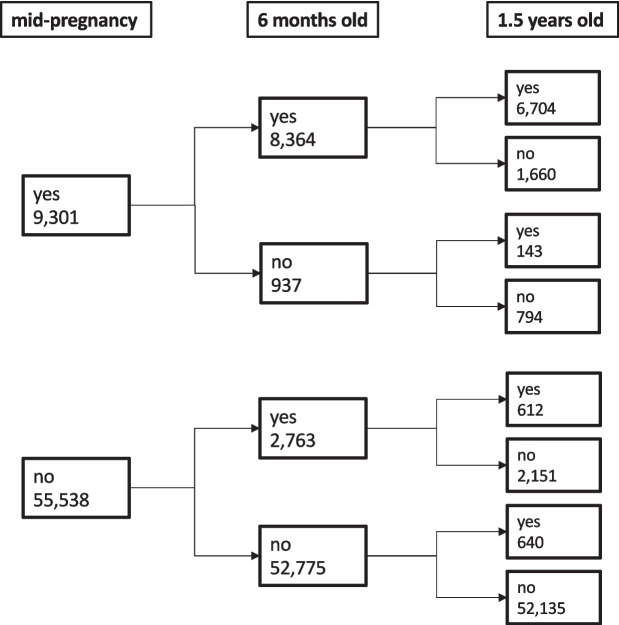
Change pattern of time-varying exposures of pet keeping. The numbers correspond to the number of people

Figure 3 shows the pattern of pet-keeping status over time. The number of respondents in each category varied depending on the pet-keeping pattern, with a particularly small number (n=143) in the “keeping in the mid-pregnancy, not keeping at 6-months-old, and keeping at 1.5-years-old” group. Although not shown in the figure (Additional file 1, Table A2), the proportion of those who had a pet mid-pregnancy as well as when the child was 6 months old was approximately 90% (8,364/9,301 respondents), and the proportion of those who neither had a pet mid-pregnancy nor when the child was 6 months old was approximately 95% (52,775/55,538 respondents). Thus, pet ownership mid-pregnancy and when the child was 6 months old was generally consistent. Pet-keeping at other time-point combinations tended to be similar, but 25% (2,454/9,301) to 35% (3,811/11,127) respondents were pet-owners mid-pregnancy and when the child was 6 months old, but not when they were 1.5 years old.

Additional file 1, Figure A1 shows the actual time point for each questionnaire. The mode of filling in questionnaires was as follows: at the time point of the 1.5-year-old’s questionnaire, 18 months ago; at the time point of the 6-month-old’s questionnaire, 30 months ago, and at the time point of the mid-pregnancy questionnaire, 39 months ago. This corresponds with a large number of participants, who responded without delay (for example, 3 years 0 months – 1.5 years 0 months = 18 months ago, corresponds to the mode at the time point of the response to the 1.5 year old’s questionnaire). Time points varied by approximately ±3 months of the mode at any time point.

#### Impact of pet-keeping over time on the incidence of childhood wheezing

Table 5 shows the results of the analysis by Single Model and Multi Model. In the Single Model, the adjusted odds ratio for keeping a pet compared to the no-pet group was significantly greater than 1 at all time points. The odds ratios for keeping a pet mid-pregnancy and when the child was 6 months and 1.5 years old were 1.13, 1.14, and 1.15, respectively, showing little change. The Multi Model showed a decrease in point estimates of odds ratios compared to the Single Model at any time point of pet-keeping status; there was no significant difference between the pet-keeping and non-pet-keeping groups. The 95% confidence intervals were also wider than in the Single Model.

**Table 5:** Estimation results of exposure effects by single model and multi model

**Pet ownership**	**Category (/ reference)**	**Wheezing in the last 12 months at 3 years old: odds ratio (95% CI)**	
		**Single Model**	**Multi Model**
Mid-pregnancy	Yes (/ No)	1.13 ( 1.07, 1.20 )	1.02 ( 0.92, 1.14 )
6 months old	Yes (/ No)	1.14 ( 1.08, 1.20 )	1.08 ( 0.98, 1.19 )
1.5 years old	Yes (/ No)	1.15 ( 1.08, 1.22 )	1.06 ( 0.96, 1.17 )

Adjustment variables: previous delivery, weeks of pregnancy at delivery, planned/emergent cesarean delivery, weight at birth, child’s sex at birth, annual household income, frequency of cleaning the living room floor with a vacuum cleaner, frequency of cleaning the bedroom floor with a vacuum cleaner, family members' smoking after the baby was born within 1 month of birth, mother’s allergy and ear-nose-throat disease (bronchial asthma), Study Areas

**Fig. 4:**
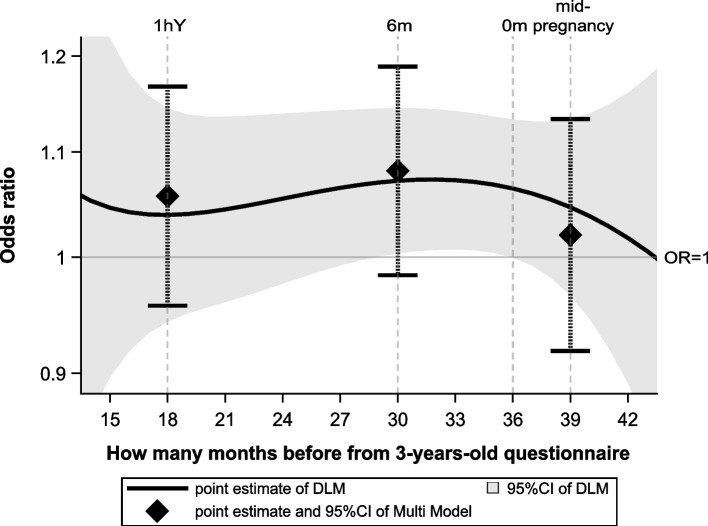
Odds ratio of pet-keeping exposure and onset of wheezing at age 3 years by DLM. For comparison, the point estimates and 95% confidence intervals of the odds ratios estimated by the Multi Model are shown for the time point of the mode of each questionnaire response. The 95% confidence interval for the DLM is narrower than that for the Multi Model. The 95% confidence intervals were widened at the two ends of the figure (from approximately 15 to 18 months and from approximately 39 to 42 months) due to a lack of available data

The results using the Multi Model and DLM are shown in Fig. 4. The analysis using DLM showed a significant increase in the odds for wheezing between lags 30 to 36. This corresponds to critical windows between the ages of 0 and 6 months, considering that most of the 3-year-old questionnaires were answered at the age of 3 years and 0 months. During that periods, the highest ORs were 1.07 (95% confidence interval, 1.01 to 1.14 ; lags 31 to 34, which corresponds to the ages between 2 and 5 months).

## Discussion

Overall, in the simulation experiments, DLM outperformed the other models in high correlation scenarios, as indicated by the MSE. The DLM analysis of the JECS data showed a statistically significant difference during the 0-6 months’ period, suggesting that this may be the critical windows.

Simulation experiments showed that the Single Model, which estimates the effects of exposures at multiple time points separately, could estimate the true values without bias only if there is no correlation between exposures, but if there is large correlation, the estimates will be biased. The Multi Model, in which exposures at multiple time points were simultaneously entered into the model, showed that, on average, the true value could be estimated without bias, but that the accuracy of the estimated exposure effects deteriorates as the correlation among exposures increases. These results are consistent with previous studies [23, 24]. The DLM, which models the estimates at each time point as a function of the time point, showed that when the shape of the true regression coefficient can be represented by a polynomial function of DLM (Scenarios 1 and 2), the true value can be estimated without bias and with small variance even when the correlation between exposures is high; when the true shape cannot be represented by the DLM (Scenario 3), the time where the peak of the true value exists can be captured with a good precision, although some bias is introduced. The MSE, which evaluates bias and variance simultaneously, indicated that when the correlation between exposures is high, it is more useful to use DLM than to model multiple exposures simultaneously. In studies where researchers want to use different exposure variables for each pregnancy trimester, there is also published result [44] that suggest the same problem as the Single and Multi models in this study. To address this issue, a study [45] have conducted sensitivity analysis, but the DLM may be useful.

The three analyses of the JECS data yielded different results regarding pet ownership and the onset of wheezing in 3-year-olds. Logistic regression with multiple time points of exposure modeled separately showed significant differences at all time points, that with multiple time points of exposure modeled simultaneously showed no significant differences at all time points, and that with DLM showed significant differences only from 0 to 6 months. The results of logistic regression with multiple time points of exposure modeled separately are likely to be strongly influenced by other time points [23], as confirmed by the simulation experiment; thus, it is difficult to conclude that pet ownership at all time points associates with wheezing onset. Moreover, as confirmed by the simulation experiment, logistic regression, in which exposures at multiple time points with large correlations were simultaneously included in the model, may have increased the exposure effect estimation error; consequently, no significant exposure effect was observed. Conversely, the DLM estimated smaller bias than the Single Model and smaller variance than the Multi Model; thus, 0–6 months was considered the critical windows. In our study, we considered using simple functions in DLM, and in practice, when attempting to estimate using splines in DLM with our data, we were unable to achieve stable estimates. Regarding the determination of critical windows, a Bayesian method has been proposed to estimate the regression coefficient for time-varying exposure by separating it into two components: one related to the outcome and the other to determine whether the time is a critical window or not [22]; however, that study used Gaussian processes instead of DLM to address correlations between exposures, and further research is warranted.

Our findings suggest a potential harm of pet ownership on children's asthma, aligning with studies indicating harmful possibilities [15], contradicting research that shows negligible effects [46] or claims benefits [13, 47]. When interpreting our results in a biological context, several prior studies [48, 49] suggesting that sensitization to allergens is associated with asthma may be useful. It is known that many inhaled allergens activate the airway epithelium through pathways such as PAR, leading to type 2 inflammation [50]. Another study [51] also suggests that sensitization to perennial allergens like cat and dog dander by the age of 3 influences a decline in respiratory function and an increase in airway hyperresponsiveness during school age. Based on these, our results indicate a potential connection between early sensitization to inhaled allergens - particularly within the first 6 months of life - and the exacerbation of wheezing due to type 2 inflammation. As stated in the Introduction, one of the objectives of identifying critical windows is to provide feedback to biological research with our findings, so it's crucial to recognize that there are limitations to the explanations we have attempted. The strength of the data is that JECS participants are recruited in large numbers from all over Japan. A comparison of the basic demographics of the JECS participants with the 2013 demographic statistics reported similar proportions of singleton births, full-term births, sex ratio, and cesarean sections, as well as the distribution of age at birth for the mother and birth weight of the child [52]. Therefore, as a strength of our study, our results may have high generalizability to the Japanese population and we were able to adjust for many potentially confounding environmental, genetic, and social variables because the JECS is a large birth cohort study of 100,000 pairs of children and parents. Furthermore, our study conducted analyses that considered the correlations between exposure variables and analyzed the time-varying pet ownership status without categorizing it. On the other hand, some studies excel by using data on the concentration of substances in the body related to exposure [46] or by utilizing outcomes [13, 47] from older age groups [14]. Regardless, further investigation, including biological research, is necessary.

Our study has some limitations. First, current research has proposed DLMs that use a spline function of time points for the constraints [26], or that simultaneously estimate the delayed effects and the effect of explanatory variables [53], but our study is not directly comparable to such complex models. Second, in this study, critical windows were determined by the 5% significance level, but the determination was strongly influenced by the study size, and we might not be able to accurately identify the critical windows.

## Conclusion

In this study, the results of the simple DLM for time-varying dichotomous exposures indicated an enhanced precision in estimating exposure effects, as opposed to the simultaneous analysis of all exposure variables.

Applying DLM to the JECS data, our findings suggest that the practice of keeping pets from birth until the age of 6 months might be correlated with the onset of wheezing by the age of 3 years.

### Supplementary Information

Supplementary Material 1. 

## Data Availability

No datasets were generated or analysed during the current study.

## Abbreviations

DLM: Distributed lag model
EmpSE: Empirical standard error
MSE: Mean squared error
JECS: The Japan Environment and Children’s Study
ISAAC: The International Study of Asthma and Allergies in Childhood
