# Supplementary material for: Association of longitudinal pet ownership with wheezing in 3-year-old children using the distributed lag model: the Japan Environment and Children’s Study
Source: Environ Health. 2024 Jun 6;23:53. doi: 10.1186/s12940-024-01087-x (PMC11155167; doi:10.1186/s12940-024-01087-x)
Supplement: Supplementary file 1 — Supplementary Material 1. [file 12940_2024_1087_MOESM1_ESM.pdf]

Association of Longitudinal Pet Ownership with Wheezing in 3-Year-Old Children Using the Distributed Lag Model: The Japan Environment and Children's Study

Web Appendix

Appendix 1. Details of JECS data analysis.

Web Tables

Table. A1: Variables used for covariates.

Variables	Questionnaire timing	Category / Additional information
Previous delivery	at study enrollment	Yes, no
Weeks of pregnancy at delivery	at delivery	premature birth (22-36 weeks),<br>full-term birth (37-41 weeks), others
Planned/emergent cesarean delivery	at delivery	Yes, no
Weight at birth	at delivery	$<$ 2,500 g, $\geq 2{,}500$ g
Child's sex at birth	at delivery	male, female
Annual household income	mid-pregnancy	$< 4, \geq 4 - 6, \geq 6$ million JPY
Frequency of cleaning the living room floor with a vacuum cleaner	mid-pregnancy	average throughout the year;<br>categorized as every day, once a week and more,<br>less than once a week
Frequency of cleaning the bedroom floor with a vacuum cleaner	mid-pregnancy	average throughout the year;<br>categorized as every day, once a week and more,<br>less than once a week
Family members' smoking after the baby	1 month old	No one smoked (no smoking),<br>Somebody smoked but not in the presence of the baby

was born within 1 month of birth		(smoking in the absence of babies), Somebody smoked in the presence of the baby (smoking in the presence of the baby)
Mother's allergy and ear-nose-throat disease	at study enrollment	Has the mother ever been diagnosed with bronchial asthma by a physician from birth to the time of study enrollment ? (yes, no)
Study Areas	3 years old	Names of currently managed regions (organizations) (Hokkaido, Miyagi, Fukushima, Chiba, Kanagawa, Koshin (Yamanashi , Shinshu), Toyama, Aichi, Kyoto (Kyoto , Doshisha), Osaka, Hyogo, Tottori, Kochi, Fukuoka (Occupational and Environmental Health , Kyushu), South Kyushu/Okinawa (Kumamoto , Miyazaki , Ryukyu)

Table. A2: Changes in pet keeping.

		6 months old pet keeping			1.5 years old pet keeping		
		Yes people	No people	Sum	Yes people	No people	Sum
Mid Pregnancy pet keeping	Yes	8,364	937	9,301	6,847	2,454	9,301
	No	2,763	52,775	55,538	1,252	54,286	55,538
	Sum			64,839			64,839
6 months old pet keeping	Yes				7,316	3,811	11,127
	No				783	52,929	53,712
	Sum						64,839

Web Figures

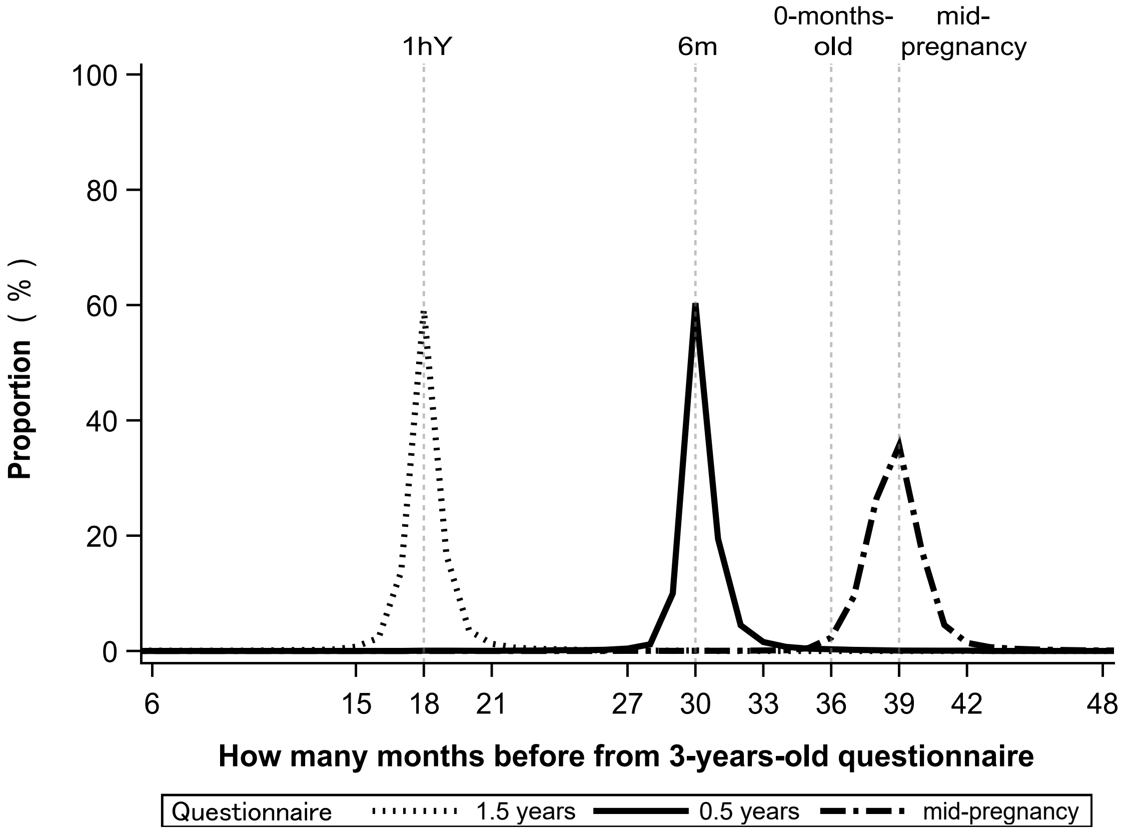

Figure. A1：Distribution of questionnaire response time.

The x-axis indicates the number of months before the 3-year-old questionnaire.

Appendix 2. Details of Simulation Experiment.

Box-and-whisker plots of the estimation results by simulation experiment for each scenario and each model are shown in Figures B1-B3 for each of the three time points at t=36, 42, and 48, for each value of γ. The box plots show that the Single Model has a large bias in the estimation and that the Multi Model captures the true values on average, but the variability of the estimates is larger than in the other models when the correlation between exposures is large.

Web Tables

Table. B1: Time point settings used in the simulation experiments.

**Time point**	**Range of time point distribution t_{ki}**	**Probability distribution of time point**
t_{1i}	$4 \leqq t_{1i} \leqq 9$	$p(t_{1i}=4)=0.10, p(t_{1i}=5)=0.15, p(t_{1i}=6)=0.45,$<br>$p(t_{1i}=7)=0.15, p(t_{1i}=8)=0.10, p(t_{1i}=9)=0.05$
t_{2i}	$10 \leqq t_{2i} \leqq 15$	$p(t_{2i}=10)=0.10, p(t_{2i}=11)=0.15, p(t_{2i}=12)=0.45,$<br>$p(t_{2i}=13)=0.15, p(t_{2i}=14)=0.10, p(t_{2i}=15)=0.05$
t_{3i}	$16 \leqq t_{3i} \leqq 21$	$p(t_{3i}=16)=0.10, p(t_{3i}=17)=0.15, p(t_{3i}=18)=0.45,$<br>$p(t_{3i}=19)=0.15, p(t_{3i}=20)=0.10, p(t_{3i}=21)=0.05$
t_{4i}	$22 \leqq t_{4i} \leqq 27$	$p(t_{4i}=22)=0.10, p(t_{4i}=23)=0.15, p(t_{4i}=24)=0.45,$<br>$p(t_{4i}=25)=0.15, p(t_{4i}=26)=0.10, p(t_{4i}=27)=0.05$
t_{5i}	$28 \leqq t_{5i} \leqq 33$	$p(t_{5i}=28)=0.10, p(t_{5i}=29)=0.15, p(t_{5i}=30)=0.45,$<br>$p(t_{5i}=31)=0.15, p(t_{5i}=32)=0.10, p(t_{5i}=33)=0.05$
t_{6i}	$34 \leqq t_{6i} \leqq 39$	$p(t_{6i}=34)=0.10, p(t_{6i}=35)=0.15, p(t_{6i}=36)=0.45,$<br>$p(t_{6i}=37)=0.15, p(t_{6i}=38)=0.10, p(t_{6i}=39)=0.05$
t_{7i}	$40 \leqq t_{7i} \leqq 45$	$p(t_{7i}=40)=0.10, p(t_{7i}=41)=0.15, p(t_{7i}=42)=0.45,$<br>$p(t_{7i}=43)=0.15, p(t_{7i}=44)=0.10, p(t_{7i}=45)=0.05$
t_{8i}	$46 \leqq t_{8i} \leqq 51$	$p(t_{8i}=46)=0.10, p(t_{8i}=47)=0.15, p(t_{8i}=48)=0.45,$

		$p(t_{8i} = 49) = 0.15, p(t_{8i} = 50) = 0.10, p(t_{8i} = 51) = 0.05$
t_{9i}	$52 \leqq t_{9i} \leqq 57$	$p(t_{9i} = 52) = 0.10, p(t_{9i} = 53) = 0.15, p(t_{9i} = 54) = 0.45,$<br>$p(t_{9i} = 55) = 0.15, p(t_{9i} = 56) = 0.10, p(t_{9i} = 57) = 0.05$
t_{10i}	$58 \leqq t_{10i} \leqq 63$	$p(t_{10i} = 58) = 0.10, p(t_{10i} = 59) = 0.15, p(t_{10i} = 60) = 0.45,$<br>$p(t_{10i} = 61) = 0.15, p(t_{10i} = 62) = 0.10, p(t_{10i} = 63) = 0.05$

Web Figures

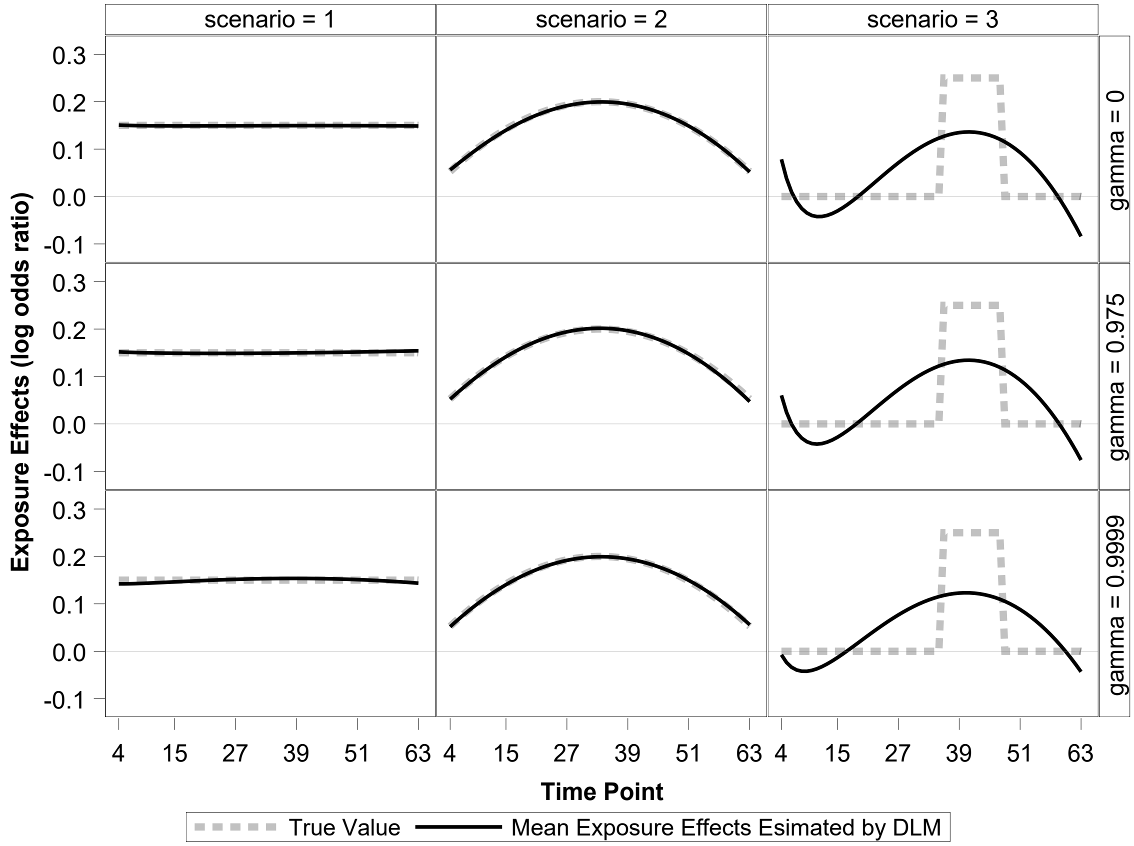

Figure. B1: Mean exposure effects by DLM per scenario and correlation coefficient (γ).

The gray dotted line: the true value. The black line: the DLM average. The gray line: reference line for β=0. Scenarios 1, 2, and 3 in order from left to right. Upper panel γ=0, middle panel γ=0.975, lower panel γ=0.9999.

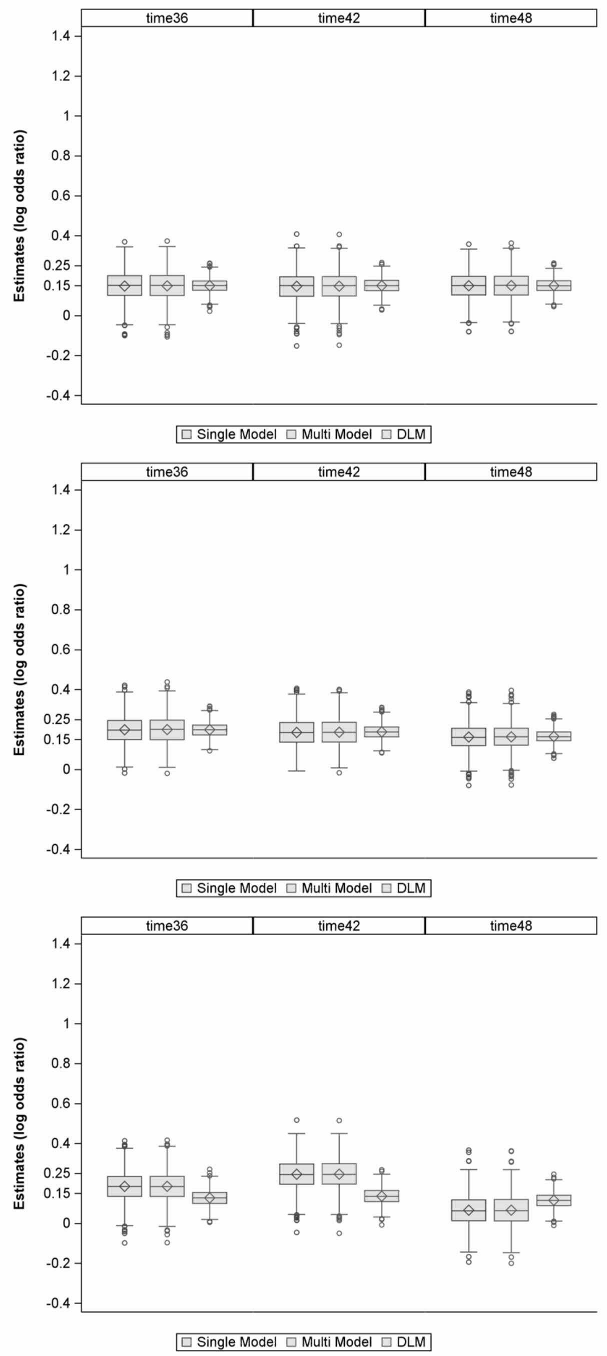

Figure. B2: Distribution of point estimates of exposure effects for scenarios 1-3 (γ=0).

Box-and-whisker plots for time=36,42,48 from left to right. Within each time, from left to right: Single model, Multi model, DLM. Scenarios 1, 2, and 3 from top to bottom.

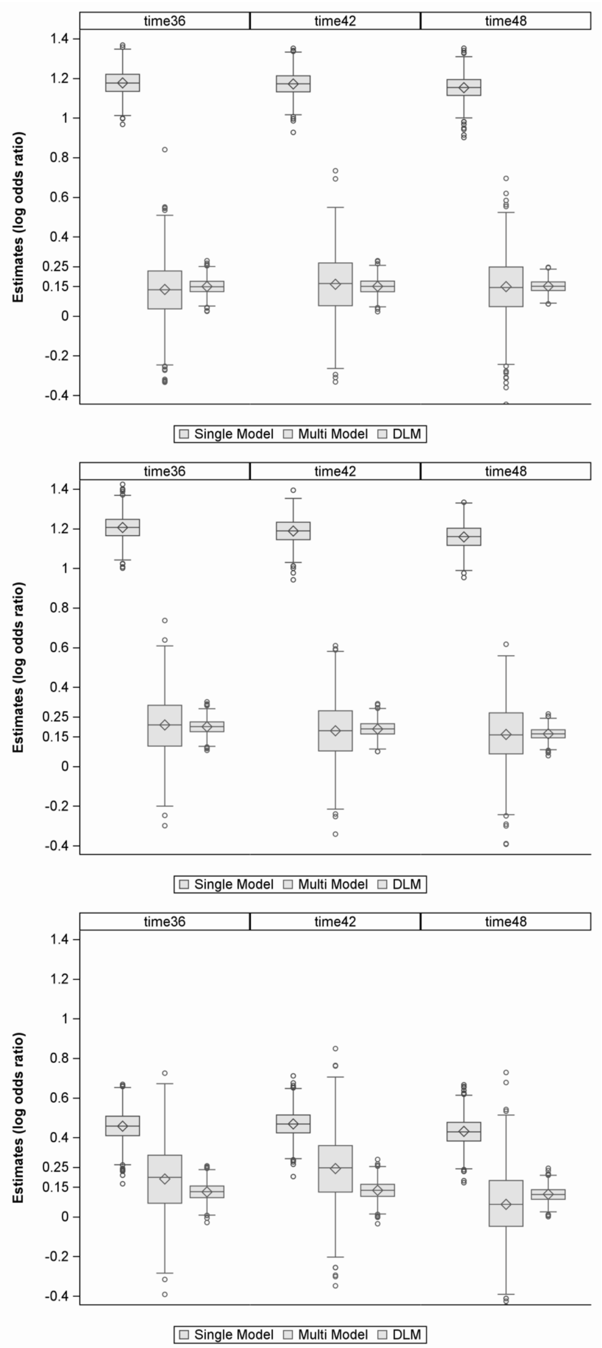

Figure. B3: Distribution of point estimates of exposure effects for scenarios 1-3 (γ=0.975).

Box-and-whisker plots for time=36,42,48 from left to right. Within each time, from left to right: Single model, Multi model, DLM. Scenarios 1, 2, and 3 from top to bottom.

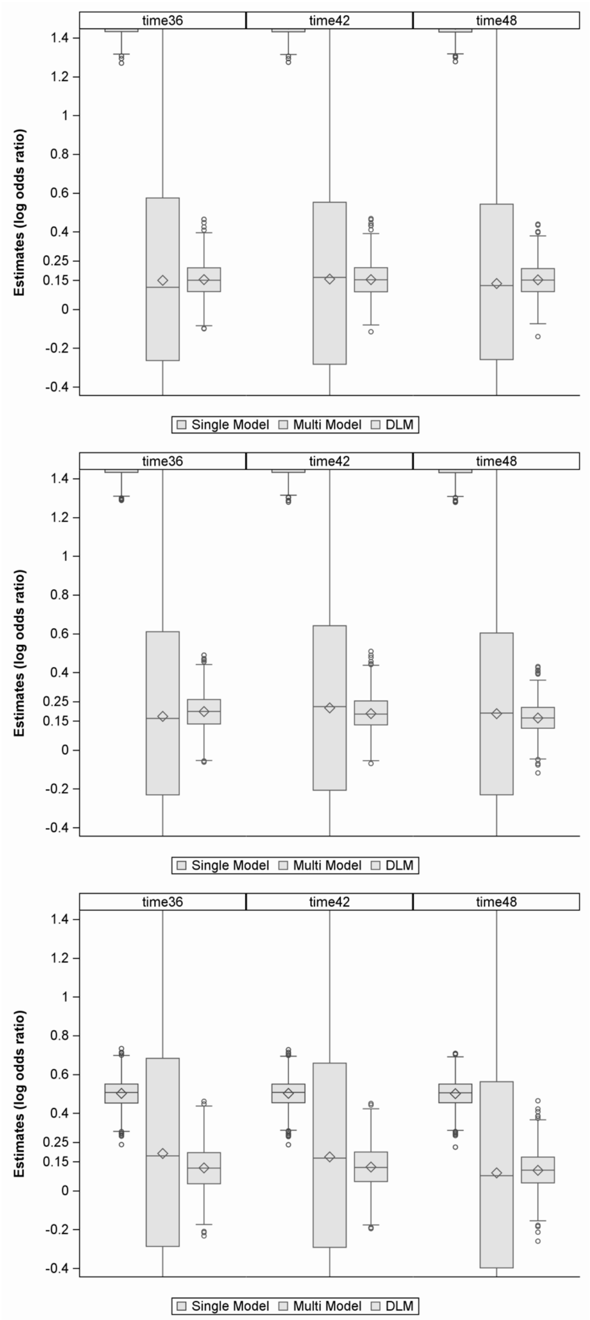

Figure. B4: Distribution of point estimates of exposure effects for scenarios 1-3 (γ=0.9999).

Box-and-whisker plots for time=36,42,48 from left to right. Within each time, from left to right: Single model, Multi model, DLM. Scenarios 1, 2, and 3 from top to bottom.

Appendix 3. Members of the JECS Group as of 2022:

Michihiro Kamijima (principal investigator, Nagoya City University, Nagoya, Japan), Shin Yamazaki (National Institute for Environmental Studies, Tsukuba, Japan), Yukihiro Ohya (National Center for Child Health and Development, Tokyo, Japan), Reiko Kishi (Hokkaido University, Sapporo, Japan), Nobuo Yaegashi (Tohoku University, Sendai, Japan), Koichi Hashimoto (Fukushima Medical University, Fukushima, Japan), Chisato Mori (Chiba University, Chiba, Japan), Shuichi Ito (Yokohama City University, Yokohama, Japan), Zentaro Yamagata (University of Yamanashi, Chuo, Japan), Hidekuni Inadera (University of Toyama, Toyama, Japan), Takeo Nakayama (Kyoto University, Kyoto, Japan), Tomotaka Sobue (Osaka University, Suita, Japan), Masayuki Shima (Hyogo Medical University, Nishinomiya, Japan), Hiroshige Nakamura (Tottori University, Yonago, Japan), Narufumi Suganuma (Kochi University, Nankoku, Japan), Koichi Kusuhara (University of Occupational and Environmental Health, Kitakyushu, Japan), and Takahiko Katoh (Kumamoto University, Kumamoto, Japan).
